# Measurement of gamma-ray dose rate distribution at the Kindai university reactor using the thermoluminescent properties of BeO ceramic plates

**DOI:** 10.1007/s12194-025-00981-4

**Published:** 2025-10-30

**Authors:** Leo Takahashi, Genichiro Wakabayashi, Kenichi Watanabe, Hiroki Tanaka, Takushi Takata, Akihiro Nohtomi, Kiyomitsu Shinsho

**Affiliations:** 1 Tokyo Metropolitan University, 7-2-10 Higashi-Ogu, Arakawa-ku, Tokyo, 116-8551 Japan; 2 Kindai University, 3-4-1 Kowakae, Higashiosaka-shi, Osaka 577-8502 Japan; 3 Graduate School of Engineering, Kyushu University, 744 Motooka, Nishi, Fukuoka 819-0395 Japan; 4 Kyoto University, 2 Asashironishi, Kumatori-cho, Sennan-gun, Osaka, 590-0494 Japan; 5 Graduate School of Medical Sciences, Kyushu University, 3-1-1 Maidashi, Higashi-ku, Fukuoka, 812-8582 Japan

**Keywords:** Thermoluminescence, BeO, UTR-KINKI, Gamma-ray dosimetry

## Abstract

The gamma-ray dose rate distribution at the Kindai University Reactor (UTR-KINKI) was measured using the thermoluminescent (TL) properties of beryllium oxide (BeO) ceramic plates. The reactor, operating at an extremely low thermal power of 1 W, is widely used for nuclear research, including radiation biology and detector development. In neutron-gamma mixed fields, determining the gamma-ray dose rate accurately is technically challenging due to the neutron sensitivity of conventional dosimeters. In this study, low-Na BeO ceramic thermoluminescence dosimeters (TLDs) were employed to selectively measure gamma-ray dose rates in the irradiation hole of UTR-KINKI, without the need for neutron correction. A comparative assessment was conducted using Na-doped BeO powder TLDs, and thermal neutron flux measurements were performed using a Li-glass scintillator. The results demonstrated that the height-dependent trend of the gamma-ray dose rate distribution was consistent with previous measurements obtained via paired ionization chambers. However, the absolute values of the gamma-ray dose rates measured with the BeO ceramic TLDs were approximately 10–30% higher than those determined by the paired ionization chamber. This discrepancy is likely due to neutron sensitivity considerations in previous studies. The gamma-ray dose rate at the reactor center was evaluated as approximately 24 cGy h^−1^. This study highlights the applicability of BeO ceramic TLDs for gamma-ray dosimetry in mixed radiation fields, offering a neutron-insensitive alternative for precise dose measurements in reactor environments.

## Introduction

The Kindai University Reactor (UTR-KINKI) is an extremely low-power reactor with a rated thermal power of 1 W; it is widely used not only for education and training but also for nuclear research, such as studies in radiation biology and the development of new radiation detectors [1–6]. At the center of the reactor, a central stringer (graphite block) hole is provided; this hole is typically used as an irradiation hole in standard irradiation experiments. The irradiation field is a mixed field of neutron and gamma-rays, with a maximum thermal neutron flux of 1.2 × 10^7^ cm^− 2^ s^− 1^ during operation at 1 W [7]. For gamma-ray dose measurements in such mixed fields, a paired ionization chamber [8, 9] and thermoluminescence dosimeter (TLD) [10, 11] have generally been used. Recently, research has also been conducted on gamma-ray dose measurements in mixed fields using a radio photoluminescence glass dosimeter (RPLGD) [12]. However, because most photon dosimeters are sensitive to neutrons, selectively measuring only the gamma-ray dose without neutron influence in a mixed field is technically difficult. Nonetheless, knowledge of the actual dose of gamma-rays is essential for planning research, selecting optimal irradiation fields, and evaluating data. Therefore, clarifying the characteristics of the irradiation field is important.

The irradiation field characteristics of UTR-KINKI, including the neutron flux distribution inside the irradiation hole, the neutron and gamma-ray energy spectra, and the dose rates, have been reported [13–18]. For neutron and gamma-ray dose rate measurements, a paired ionization chamber (C-CO_2_ chamber and TE-TE chamber) has been primarily used. Endo et al. [14, 15] measured the neutron and gamma-ray dose rate distributions in the height direction of the irradiation hole and reported that the dose rates at the center of the reactor were approximately 20 cGy h^− 1^ for both neutrons and gamma-rays. In addition, Ogawa et al. [18] compared the gamma-ray dose rate distributions measured using a paired ionization chamber with those measured using TLDs. In their report, the gamma-ray dose rate near the center of the reactor was approximately 26 cGy h^− 1^; thus, the exact dose rate remains unknown.

The paired ionization chamber method has been widely used as a reliable method for determining both neutron and gamma-ray doses in mixed fields. However, this measurement method requires solving a system of equations using the relative gamma-ray sensitivity and relative neutron sensitivity of each ionization chamber:

1 $$\begin{array}{c}{R}_{\mathrm{T}}={k}_{\mathrm{T}}{D}_{\mathrm{N}}+{h}_{\mathrm{T}}{D}_{\mathrm{G}}\end{array}$$

2 $${R}_{\mathrm{U}}={k}_{\mathrm{U}}{D}_{\mathrm{N}}+{h}_{\mathrm{U}}{D}_{\mathrm{G}}$$

where $\:{R}_{T}$ and $\:{R}_{U}$​ are the responses of the TE–TE and C–CO₂ chambers, respectively; $\:{h}_{T}$​ and $\:{h}_{U}\:$are the relative sensitivities to gamma rays; and $\:{k}_{\mathrm{T}}$ and $\:{k}_{U}$ are the relative sensitivities to neutrons, respectively; $\:{D}_{\mathrm{G}}$ and $\:{D}_{N}$​ are the gamma-ray dose and neutron dose. Although $\:{h}_{T}$​ and $\:{h}_{U}$​ are typically close to 1, allowing for accurate calculations, $\:{k}_{\mathrm{U}}$ strongly depends on the neutron energy, making precise calculations difficult [19]. Consequently, the calculated gamma-ray dose is inevitably influenced to some extent by neutrons. Similarly, gamma-ray measurement methods that involve using a TLD or RPLGD in neutron fields are also affected by neutrons.

In the present study, we used low-Na plate-type BeO ceramic TLDs to measure the gamma-ray dose rate distribution in the irradiation hole of UTR-KINKI. BeO-based TLDs have a small neutron capture cross-section and excellent tissue equivalence, making them well-suited for measuring absorbed dose in biological tissue. Owing to these advantages, Na-doped BeO powder TLDs have been used for gamma-ray dosimetry in neutron and gamma-ray mixed fields. However, their production has been discontinued due to concerns over the risk of toxic BeO powder dispersion in the event of quartz glass tube breakage. Therefore, we adopted a BeO ceramic TLD, which eliminates this risk and is increasingly being recognized as a viable substitute for conventional BeO powder TLDs. Moreover, through irradiation experiments at the heavy-water neutron irradiation facility at the Kyoto University Reactor (KUR) (maximum thermal neutron flux of 5 × 10^9^ cm^− 2^ s^− 1^), researchers have demonstrated that the low-Na BeO ceramic TLD can measure gamma-ray dose rates in neutron and gamma-ray mixed fields without correction for the effects of neutrons [20]. For comparison, we also conducted gamma-ray dose measurements using Na-doped BeO powder TLDs. In addition, thermal neutron flux distribution measurements in the irradiation hole were performed using a Li-glass scintillator, and the irradiation field characteristics were clarified by comparing these measurements with the measured gamma-ray dose rate distribution.

## Materials and methods

### BeO TLD

#### BeO ceramic TLD

**Fig. 1:**
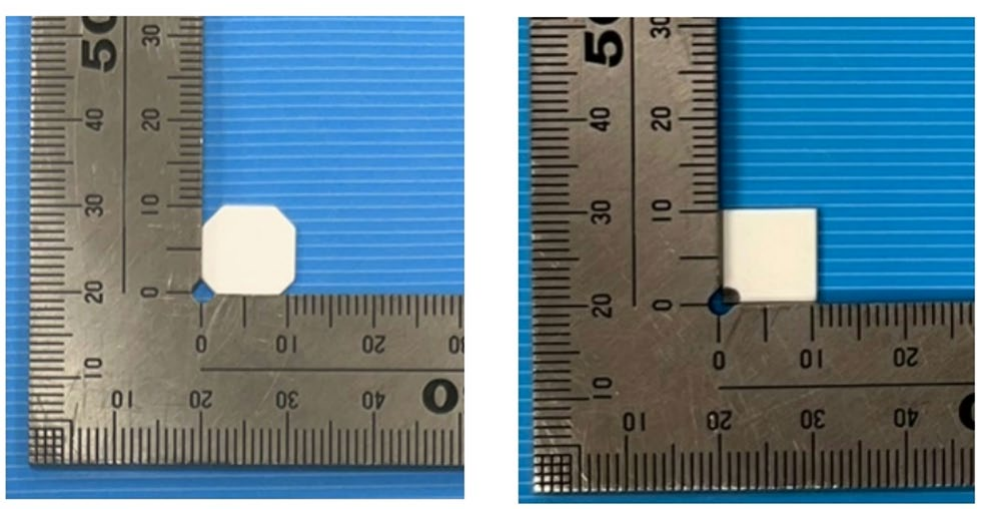
The BeO ceramic TLDs (left: Sample **A**; right: Sample **B**)

A plate-type BeO ceramic TLD (Thermalox 995, Materion) was used. Its composition was > 99.5 wt% BeO, with its other components listed in Table 1. The effective atomic number was 7.13, and the density was 2.85 g cm^− 3^. Compared with the BeO powder TLD, it contained less Na. As shown in Fig. 1, two different Samples of the BeO ceramic TLD were used, with plate dimensions of 11 mm × 11 mm × 0.7 mm for Sample A and 10 mm × 10 mm × 0.7 mm for Sample B. The Samples did not differ with each other in composition, manufacturing process, or TL characteristics. However, because the difference in physical dimensions may affect the dose response, the samples were described separately as A and B. In this study, individual TL-dose conversion tables were created for each sample; therefore, it was not strictly necessary to separate them. However, they are distinguished here to clarify the difference in shape.

**Table 1:** Impurities in the BeO ceramic TLDs, and their concentrations

Element	Si	Mg	Na	Al	Fe	Ca	Zn
Concentration (wt%)	0.1861	0.0922	0.0173	0.0046	0.0032	0.0031	0.0020

#### BeO powder TLD

**Fig. 2:**
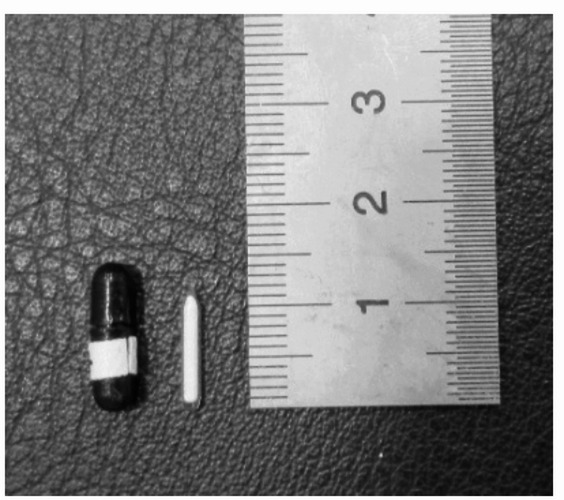
BeO powder TLD

The BeO powder TLD (UD-170LS, Panasonic) in which powdered beryllium oxide (BeO) is encapsulated in a quartz glass tube was used (Fig. 2) [21]. The elemental composition and concentrations of the TLD material are listed in Table 2. The effective atomic number was 7.6, and the thermal neutron capture cross-section of ^9^Be is 8.49 mb; however, a small amount of ^23^Na, which has a thermal neutron capture cross-section of 531.6 mb, was also added to enhance the thermoluminescence (TL) efficiency [22].

**Table 2:** Composition and concentration of BeO powder TLD [23]

Element	Be	O	Na	S
Concentration (wt%)	35.326	63.634	0.580	0.461

### Li-glass scintillator

**Fig. 3:**
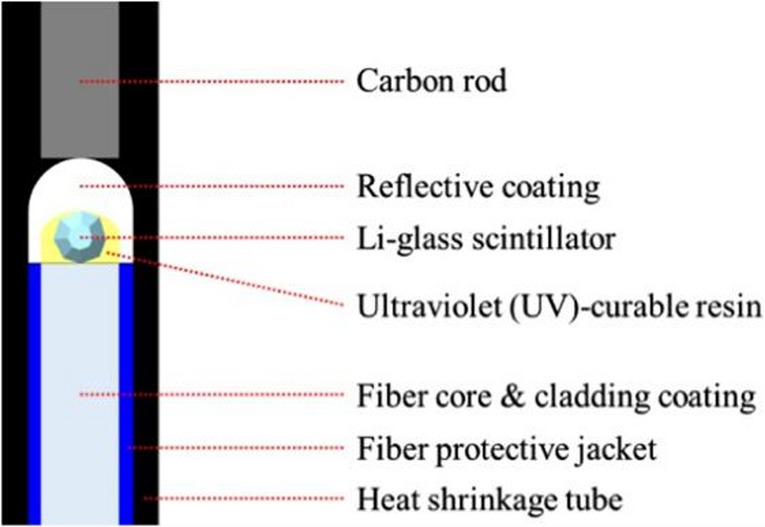
The cross-sectional structure of the optical-fiber-based neutron detector [25]

**Fig. 4:**
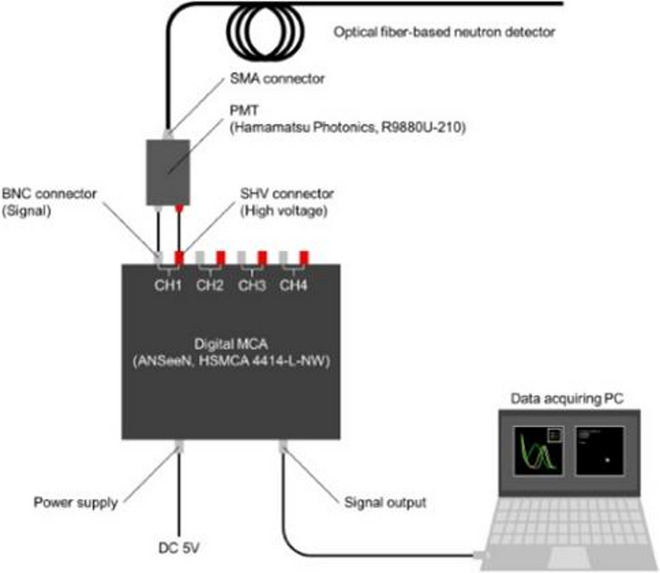
The signal processing circuits of the optical-fiber-based neutron detector system [25]

A Li-glass scintillator was used as the neutron monitor (Fig. 3) [24, 25]. This detector induces scintillation from the alpha particles and tritons produced by the ^6^Li(n, t)α reaction, and the scintillation light is transmitted through an optical fiber to a photomultiplier tube (Fig. 4). The scintillator can be used as a real-time neutron monitor, and it has been shown to exhibit high output linearity with respect to the thermal neutron flux, up to approximately 4.5 × 10^9^ n cm^− 2^ s^− 1^ [25].

### Calibration of BeO TLD

#### BeO ceramic TLD

To convert the TL intensity of the BeO ceramic TLD into a dose, we constructed a TL–dose conversion table for each Sample. For this graph, 6 MV X-rays were irradiated using a linear accelerator (Versa HD, Elekta). The irradiation field was 10 cm × 10 cm, and the doses were 0.1, 0.2, 0.3, 0.5, 0.7, 1.0, and 2.0 Gy. The average dose rate of the X-rays irradiated to the BeO ceramic TLDs was 3.8 Gy min^− 1^. Three BeO ceramic TLDs were used for each dose. The source–surface distance was set to 90 cm, with a 10 cm-thick tough water phantom placed above the TLDs and a 5 cm-thick tough water phantom placed below to sandwich the TLDs.

After irradiation, the TLDs were stored under refrigeration at approximately 5 °C to minimize fading, and TL measurements were performed between 12 and 48 h post irradiation. In this study, the TL intensity of the BeO ceramic TLD was converted into dose using the approximation equation of the TL–dose conversion table.

#### BeO powder TLD

The TL–dose calibration for the BeO powder TLD was conducted using a ^60^Co gamma-ray irradiation unit at the Institute for Integrated Radiation and Nuclear Science, Kyoto University (KURNS). The TLDs were placed at a depth of 10 cm in a 20 cm × 20 cm × 20 cm water phantom and irradiated with a dose of 300 mGy at a dose rate of 60 mGy min^− 1^. An ionization chamber (C-110, Applied Engineering) was used as the reference dosimeter. TL measurement was performed 1 h after irradiation. The conversion from TL (TLD reader output) to dose was based on the assumption of a linear response. The proportionality constant was determined from the average of three measurements, and the intercept was assumed to be zero.

In addition, a fading test was conducted for the BeO powder TLD under the same irradiation conditions as those used for TL–dose calibration. TL measurements were performed at 3.2 h, 5.5 h, 17 h, 2 days, 4 days, and 7 days after irradiation. The TLD reader output at each time point was evaluated relative to that measured 1 h after irradiation.

### Annealing

When TLDs are reused, annealing must be performed beforehand. Annealing is a process in which the TLDs are heated to a high temperature to release any electrons and holes remaining in the trapping centers that were not read out during previous measurements, thereby resetting the detector. To improve measurement accuracy, all TLDs were annealed prior to the experiments.

In this study, the BeO ceramic TLDs were annealed by heating at 1000 °C for 30 min using an electric furnace and controller (SAH0869, Sakaguchi Electric Heaters). The BeO powder TLDs were annealed by heating at 450 °C for 60 min using an electric furnace (NHK-120H-2, Nitto Kagaku).

### TL measurement system for the BeO TLD

#### BeO ceramic TLD

**Fig. 5:**
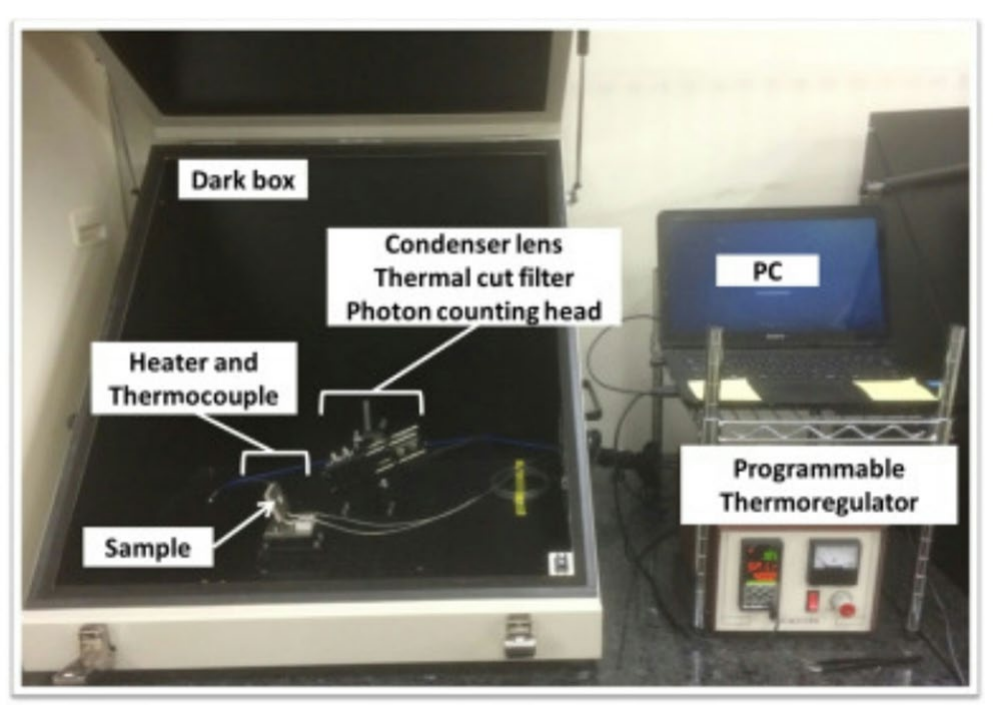
TL measurement system for the BeO ceramic TLD [26]

The TL measurement system for the BeO ceramic TLD mainly consisted of a dark box, a PC, and a programmable thermoregulator (Fig. 5) [26]. Inside the dark box, a brass heater served as a sample stage. The TL emitted during heating was collected using a condenser lens and detected by a photon counting head (H11890-210, Hamamatsu Photonics), with the data recorded on a PC. The heater temperature was controlled using a programmable thermoregulator (SCR-SHQ-A, Sakaguchi Electric Heaters), and the temperature was increased from room temperature to 400 °C at a heating rate of 0.13 °C s^− 1^. The TL intensity of the BeO ceramic TLD was defined as the integrated luminescence in the temperature range from 50 °C to 400 °C.

#### BeO powder TLD

**Fig. 6:**
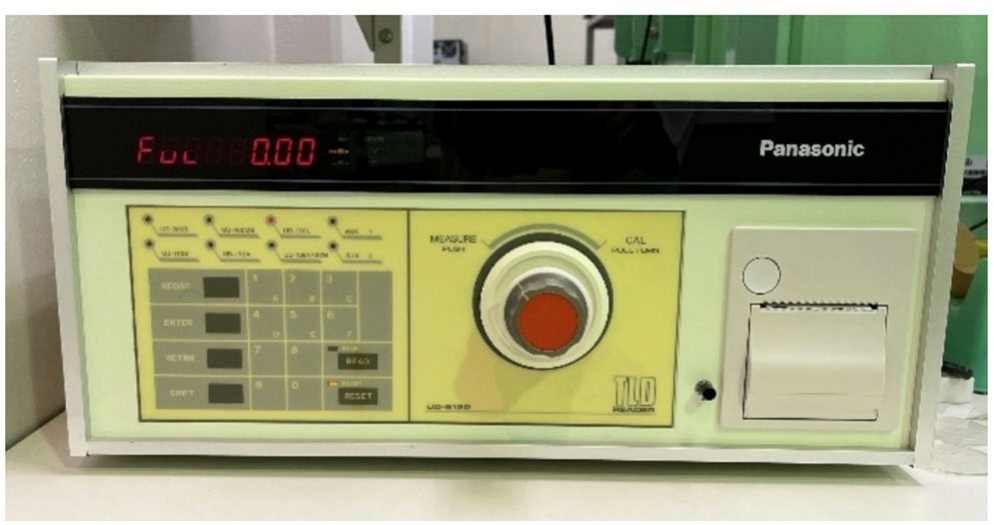
The TLD reader (UD-512P, Panasonic)

The TL measurements for the BeO powder TLD were performed using a commercial TLD reader (UD-512P, Panasonic) shown in Fig. 6. The reader was set to a heater temperature of 421 °C, with an integration time of 6 s for TL signal acquisition.

### Irradiation experiments

**Fig. 7:**
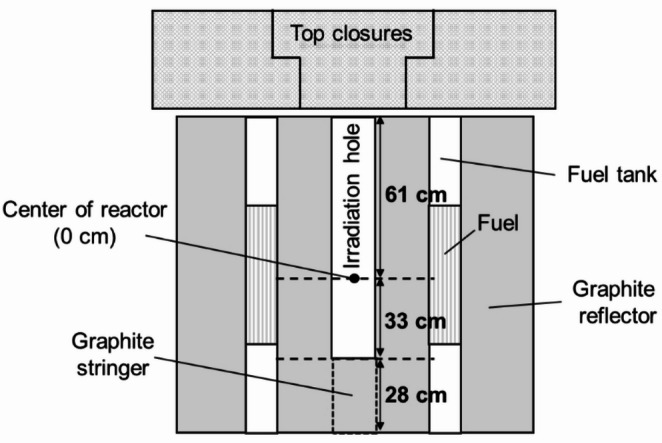
Cross-sectional view of the UTR-KINKI

As shown in Fig. 7, the UTR-KINKI consists of two fuel tanks separated by a graphite reflector; the tanks contain both fuel and a moderator (light water). At the center of the reactor, there is a central stringer made of a 9.6 cm × 9.6 cm square graphite block with a length of 122 cm, which can be removed to serve as an irradiation hole. In this study, the central stringer was partially withdrawn, leaving the lower 28 cm in place to serve as the irradiation hole. This irradiation hole extends from − 33 cm to 61 cm in height, with the reactor center defined as 0 cm. The irradiation time was defined as the duration from the moment the reactor reached a 1 W critical state until it was shut down by scram.

#### Gamma-ray dose rate distribution measurement

**Fig. 8:**
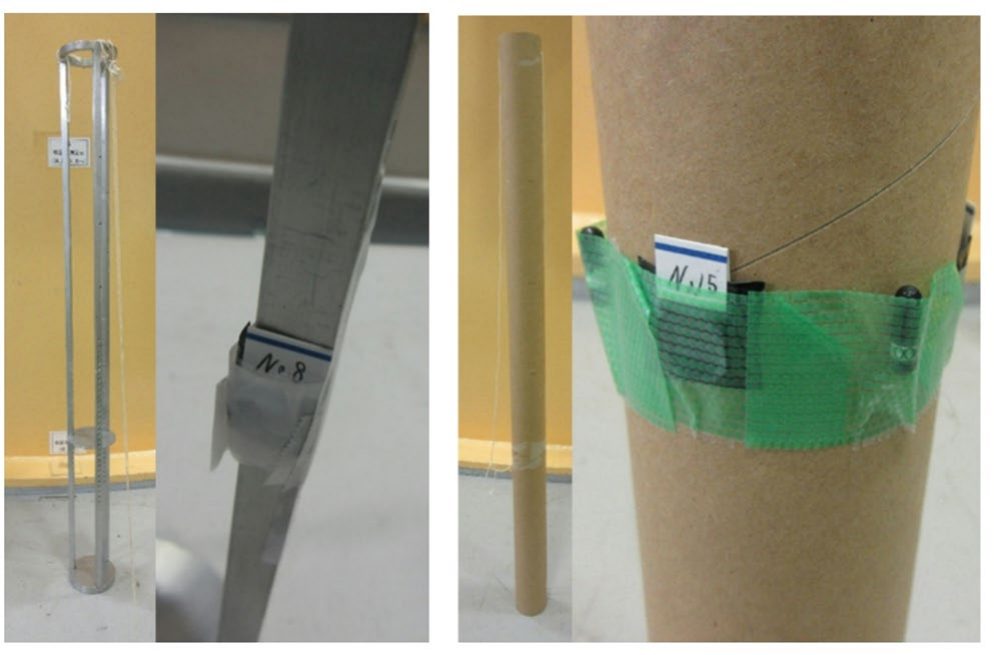
Placement of irradiation samples (left: Al frame; right: paper tube)

In addition to the BeO ceramic TLD, which was shielded from light by a thin, soft, polyvinyl chloride bag, the BeO powder TLD was used as an irradiation sample for comparison. The irradiation samples were placed in an aluminum frame (diameter: 9 cm, height: 94 cm) used in biological irradiation experiments and a paper tube (diameter: 6 cm, height: 97 cm), both of which were assembled to match the size of the irradiation hole (Fig. 8). We investigated the effect of these two materials on the gamma-ray dose in the irradiation field. The irradiation samples were placed at the following heights: BeO ceramic TLDs at − 30, −15, 0, 15, 30, 45, and 61 cm, and BeO powder TLDs at − 30, −15, 0, 30, and 61 cm, with three samples of each type placed at each height. In addition, at the reactor center height, three BeO ceramic TLDs and six BeO powder TLDs were placed in an Al frame and a paper tube, respectively, and irradiated again under the same conditions. The irradiation time was set to 3 h at the 1 W critical state.

The BeO ceramic TLDs were stored under refrigeration at approximately 5 °C, as in the TL–dose calibration procedure, and TL measurements were conducted 2 to 4 days after irradiation. As previous studies have shown that the BeO ceramic TLD exhibits no significant fading 24 h after irradiation, no fading correction was applied in this study [27, 28]. TL measurements for the BeO powder TLDs were performed between 3 and 48 h after irradiation, and fading correction was applied.

The gamma-ray dose rate distribution measured using the BeO ceramic TLD and the BeO powder TLD was compared with that measured by Endo et al. [15] using a paired ionization chamber. The gamma-ray dose rates of both of the BeO TLDs were determined as the average of the values obtained for the irradiation samples placed at each height, with the standard deviation taken as the error range. In addition, the correction value for the dose contribution from thermal neutrons in the BeO powder TLD was set to (5.1 ± 0.8) × 10^− 14^ Gy cm^2^ on the basis of measurements conducted at Japan Research Reactor-4 (JRR-4) [29]. The error range of the paired ionization chamber for the gamma-ray dose ($\:{D}_{\mathrm{G}}$) was calculated using the same method that Nohtomi et al. used to determine the uncertainty of the neutron dose ($\:{\varDelta\:D}_{\mathrm{N}}$) of the paired ionization chamber (i.e., using the error propagation formula) [30, 31]. First, $\:{\varDelta\:D}_{G}^{2}$​ was calculated as follows:

$$\:{\varDelta\:D}_{G}^{2}={\left[\frac{{R}_{\mathrm{U}}-{h}_{\mathrm{U}}{D}_{\mathrm{G}}}{{h}_{\mathrm{U}}{k}_{\mathrm{T}}-{h}_{\mathrm{T}}{k}_{\mathrm{u}}}\right]}^{2}{{\varDelta\:k}_{\mathrm{T}}}^{2}+{\left[-\frac{{R}_{\mathrm{T}}-{h}_{\mathrm{T}}{D}_{\mathrm{G}}}{{h}_{\mathrm{U}}{k}_{\mathrm{T}}-{h}_{\mathrm{T}}{k}_{\mathrm{u}}}\right]}^{2}{{\varDelta\:k}_{\mathrm{U}}}^{2}$$

3 $$\:\begin{array}{c}{\:\:\:\:\:\:\:\:\:\:\:\:\:\:\:\:\:\:+\left[\frac{{k}_{\mathrm{U}}{D}_{\mathrm{G}}}{{h}_{\mathrm{U}}{k}_{\mathrm{T}}-{h}_{\mathrm{T}}{k}_{\mathrm{u}}}\right]}^{2}{{\varDelta\:h}_{\mathrm{T}}}^{2}+{\left[-\frac{{k}_{\mathrm{T}}{D}_{\mathrm{G}}}{{h}_{\mathrm{U}}{k}_{\mathrm{T}}-{h}_{\mathrm{T}}{k}_{\mathrm{u}}}\right]}^{2}{{\varDelta\:h}_{\mathrm{U}}}^{2}\end{array}$$

Here, $\:{\varDelta\:D}_{\mathrm{G}}$​ is the uncertainty in $\:{D}_{\mathrm{G}}$​; $\:{\varDelta\:h}_{\mathrm{T}}$ and $\:{\varDelta\:h}_{\mathrm{U}}$​ are the uncertainties in the relative gamma-ray sensitivities $\:{h}_{\mathrm{T}}$​ and $\:{h}_{\mathrm{U}}$​; and $\:{\varDelta\:k}_{\mathrm{T}}$ and $\:{\varDelta\:k}_{\mathrm{U}}$​​ are the uncertainties in the relative neutron sensitivities $\:{k}_{\mathrm{T}}$​ and $\:{k}_{\mathrm{U}}$, respectively. The relative error, $\:{\varDelta\:D}_{\mathrm{G}}/{D}_{\mathrm{G}}$​, was then used as the error range. The paired ionization chamber measurements reported by Endo et al. [15] were performed using a TE–TE chamber (IC-17, Far West Tech.) and a C–CO_2_ chamber (IC-17G, Far West Tech.). In their calculation, $\:{k}_{\mathrm{T}}$​ and $\:{k}_{\mathrm{U}}$ were assumed to be identical to those for ^252^Cf fission neutrons, with values of $\:{k}_{\mathrm{T}}=0.98$ and $\:{k}_{\mathrm{U}}=0.08$. Therefore, for the uncertainty calculation, the gamma-ray relative sensitivities ($\:{h}_{\mathrm{T}},\:{h}_{\mathrm{U}}$) and neutron relative sensitivities ($\:{k}_{\mathrm{T}},\:{k}_{\mathrm{U}}$) of the TE-TE chamber and C-CO_2_ chamber, along with their uncertainties, were set as follows:

4 $$\:\left\{\begin{array}{c}{h}_{\mathrm{T}}=1,{\varDelta\:h}_{\mathrm{T}}/{h}_{T}=0.05\\\:{h}_{\mathrm{U}}=1,{\varDelta\:h}_{\mathrm{U}}/{h}_{\mathrm{U}}=0.05\end{array}\right.\mathrm{f}\mathrm{o}\mathrm{r}\:\mathrm{g}\mathrm{a}\mathrm{m}\mathrm{m}\mathrm{a}-\mathrm{r}\mathrm{a}\mathrm{y}\mathrm{s}$$

5 $$\:\left\{\begin{array}{c}{k}_{\mathrm{T}}=0.98,{\varDelta\:k}_{\mathrm{T}}/{k}_{T}=0.10\\\:{k}_{\mathrm{U}}=0.08,{\varDelta\:k}_{\mathrm{U}}/{k}_{\mathrm{U}}=0.20\end{array}\right.\mathrm{f}\mathrm{o}\mathrm{r}\:\mathrm{n}\mathrm{e}\mathrm{u}\mathrm{t}\mathrm{r}\mathrm{o}\mathrm{n}\mathrm{s}$$

#### Thermal neutron flux distribution measurement

**Fig. 9:**
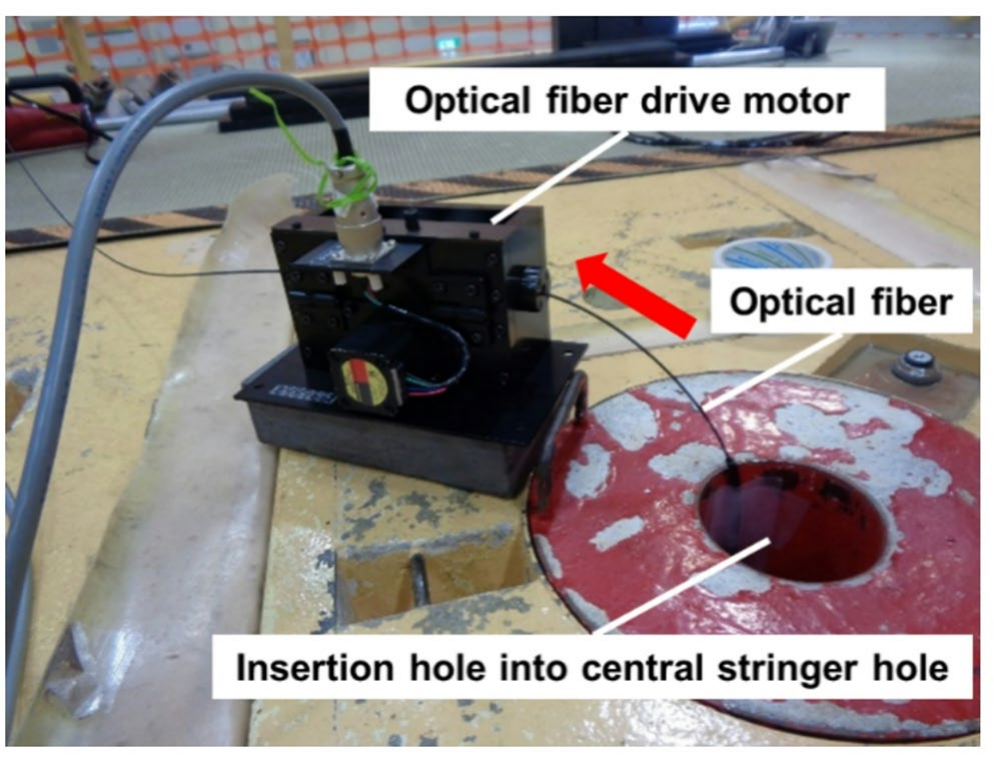
Optical-fiber drive motor

The tip of the optical fiber with a Li-glass scintillator was inserted into the irradiation hole and pulled up along the Al frame from the bottom end (− 33 cm) to the top end (61 cm) for the measurement. The scintillator was raised at a lifting speed of 0.1 m min^− 1^ using an optical-fiber driving device installed at the top of the reactor (Fig. 9). The emitted light was collected at 1 s intervals, and the measurement was performed twice. The measurement was conducted under a 1 W critical state.

## Results and discussion

### TL–dose conversion table for the BeO ceramic TLD

**Fig. 10:**
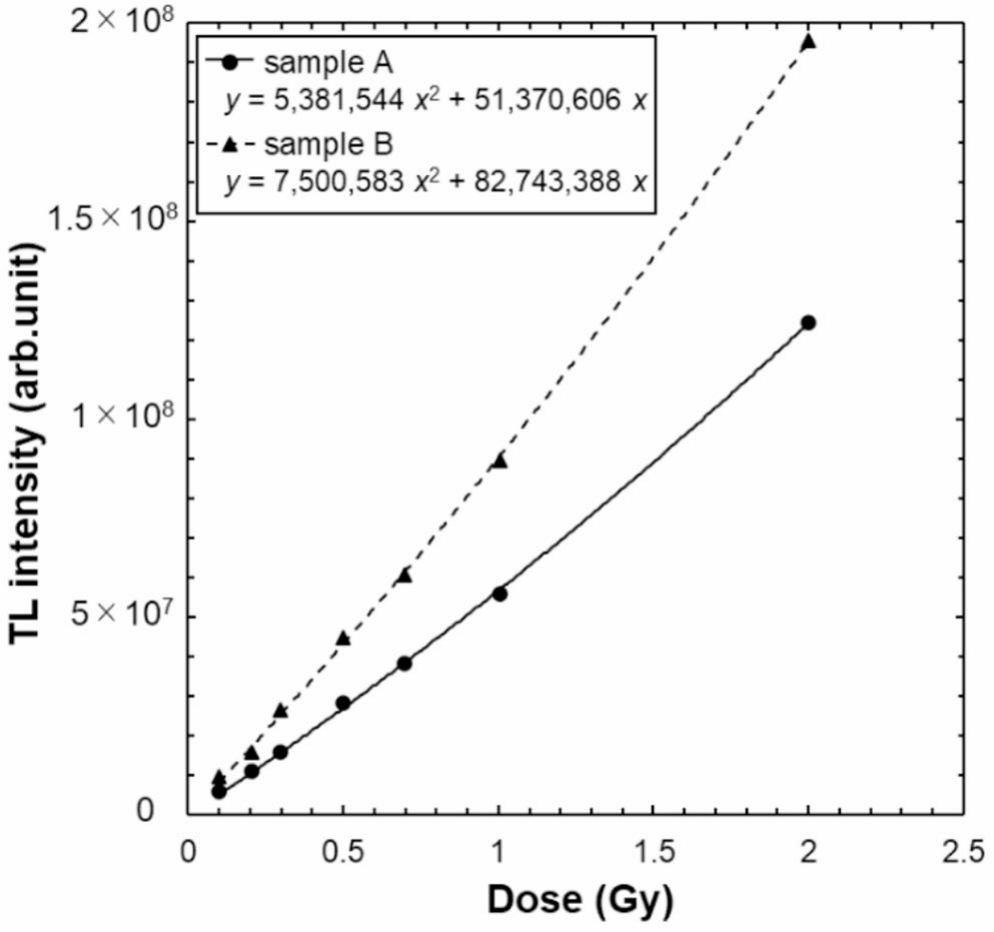
TL–dose conversion table

The TL–dose conversion table for the BeO ceramic TLD is shown in Fig. 10. The increase in TL intensity with increasing dose was approximated by a quadratic function. The coefficients of determination for the approximation equations of the two different Samples were both 0.9997, indicating excellent dose response characteristics.

### Gamma-ray dose rate distribution in the irradiation hole of UTR-KINKI

**Fig. 11:**
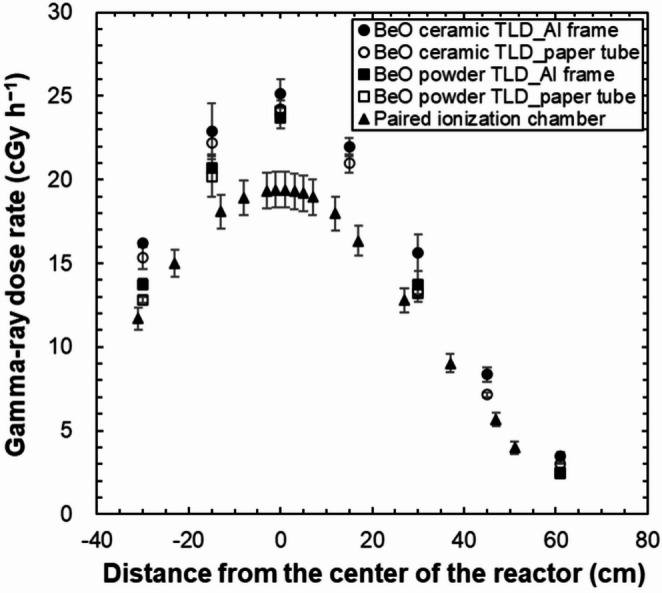
Comparison of the gamma-ray dose rate distributions measured by the BeO TLD and a paired ionization chamber

Figure 11 shows a comparison of the gamma-ray dose rate distributions measured using the BeO ceramic TLD and the BeO powder TLD in the irradiation hole of UTR-KINKI with those measured by Endo et al. [15] using a paired ionization chamber. In this study, the BeO powder TLD (UD-170LS) used for comparison contains approximately 33.5 times more sodium (Na) than the “low-Na” BeO ceramic TLD (Thermalox 995). The thermal neutron sensitivity of the BeO powder TLD, which requires a correction of approximately (5.1 ± 0.8) × 10^− 14^ Gy cm^2^ as described in Sect. 2.6.1, contributes to the observed overestimation in the gamma-ray dose rates. Given that the thermal neutron fluence near the reactor center of UTR-KINKI at 1 W for 1 h is approximately 4.0 × 10^10^ cm^− 2^, this corresponds to a correction of approximately 0.2 cGy h^− 1^. Assuming that this sensitivity arises from the Na content in the TLD, the corresponding correction for the BeO ceramic TLD used in this study is estimated to be approximately 6.1 × 10^− 3^ cGy h^− 1^, which is negligibly small. Therefore, the contribution of the trace amount of Na in the BeO ceramic TLD to thermal neutron sensitivity can be considered insignificant in this study.

The gamma-ray dose rate distributions in the irradiation hole measured by both BeO TLDs exhibited a height dependence similar to that of the rate distributions in the irradiation hole measured by the paired ionization chamber, where the highest values correspond to the center of the reactor and the values decrease with increasing distance from the center. These results are attributable to a greater neutron population near the center of the reactor, which leads to the generation of gamma-rays from fission and from interactions between neutrons and the moderator (light water). In addition, the gamma-ray dose rates measured by both BeO TLDs were found to be higher than those obtained with the paired ionization chamber at all heights, with the difference being greater closer to the center of the reactor. At the center of the reactor, the gamma-ray dose rate measured using the BeO ceramic TLD was, on average, approximately 27.8% (Al frame: ~30.2%, paper tube: ~25.4%) higher than that measured using the paired ionization chamber; the gamma-ray dose rate measured using the BeO powder TLD was approximately 23.6% (Al frame: ~22.5%, paper tube: ~24.7%) higher than that measured using the paired ionization chamber.

The gamma-ray dose rate distribution measured by the BeO ceramic TLD was evaluated to be approximately 10–30% higher than that measured by the paired ionization chamber, depending on the height. When a dosimeter sensitive to neutrons is used for dose measurements in a mixed field of neutrons and gamma-rays, the sensitivity to neutrons should ideally be calculated using the neutron spectrum of the actual irradiation field to be measured. However, in practice, calculating the sensitivity to neutrons for each irradiation field is difficult. Therefore, Endo et al. [14, 15] assumed that the sensitivity of the paired ionization chamber to neutrons is the same as that for ^252^Cf fission neutrons in their measurements. The energy spectrum of ^252^Cf fission neutrons is dominated by fast neutrons, with an average energy of approximately 2 MeV. In contrast, the neutron spectrum in the UTR-KINKI is dominated by thermal neutrons. When a dosimeter sensitive to neutrons is used in a mixed field, its response varies depending on the neutron energy spectrum. Therefore, a mismatch between the assumed and actual neutron spectra may introduce uncertainty in the evaluation of the gamma-ray dose. In this study, using the method of Nohtomi et al. [31], the uncertainty $\:{\varDelta\:D}_{\mathrm{G}}/{D}_{\mathrm{G}}$ of the gamma-ray dose determined by the paired ionization chamber was evaluated to be approximately 5.5–9.3%. However, this estimation does not include the uncertainty of the ionization current measurement. In this study, the BeO ceramic TLD with negligible sensitivity to neutrons was used, thereby avoiding such uncertainties. This difference in neutron sensitivity is considered to be one of the contributing factors to the discrepancy observed between the gamma-ray dose rates evaluated using the paired ionization chamber and the BeO ceramic TLD. In addition, Ogawa et al. [18] compared the gamma-ray dose rate distribution of an irradiation hole measured using a paired ionization chamber with that measured using TLDs. The two types of TLDs used in the measurements were a CaSO_4_-based TLD (UD-110 S) and a BeO-based TLD (UD-110 A). In the present report, the gamma-ray dose rate distribution was evaluated in the range from approximately − 28 cm to 34 cm from the center of the reactor and the results from the paired ionization chamber and TLDs were found to agree well within this range. The gamma-ray dose rate near the center of the reactor was evaluated to be approximately 26 cGy h^− 1^, and its distribution was found to be close to that measured using the BeO ceramic TLD. However, for measurements using the two types of TLDs (CaSO_4_-based TLD (UD-110 S) and BeO-based TLD (UD-110 A)), the sensitivity to neutrons, the disturbance of the neutron field due to the use of ^6^LiF cells, and the energy dependence for gamma-rays must be considered, and these uncertainties are included. In addition, another possible cause of the difference in measurements between the present study and the previous studies is the temporal variation of the reactor power at UTR-KINKI. The reactor power at UTR-KINKI is checked once per year, during a regular inspection, by measurement of the thermal neutron flux using the gold foil activation method. This inspection is considered acceptable within 30% of the rated power of 1 W. Therefore, fluctuations in thermal power within ± 30% of the rated 1 W output are possible depending on the reactor operating conditions.

In previous studies, the gamma-ray dose rates in the irradiation hole of UTR-KINKI were measured using a paired ionization chamber and TLDs [14, 15, 18]. However, both types of dosimeters required consideration of neutron effects, and the precise dose rates were not clarified. In the present study, the gamma-ray dose rate distribution was measured using a BeO ceramic TLD without correcting for neutron effects and the gamma-ray dose rate at the center of the reactor was evaluated to be approximately 24 cGy h^− 1^. One possible cause of the difference in gamma-ray dose rates between this study and previous studies is the variation in the reactor power of the UTR-KINKI. However, even in the case of such changes in the irradiation field, the gamma-ray dosimetry method using a BeO ceramic TLD, which is not sensitive to neutrons, is not affected by changes in the irradiation field.

**Fig. 12:**
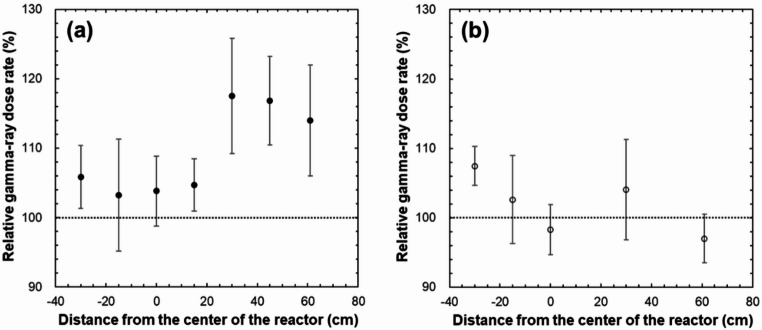
Percentage of the gamma-ray dose rate in the Al frame relative to that in the paper tube for the BeO ceramic TLD and the BeO powder TLD (a: BeO ceramic TLD, b: BeO powder TLD)

Figure 12a shows a comparison of the gamma-ray dose rates when the BeO ceramic TLD is placed in the Al frame and in the paper tube. The dose rate of the TLD placed in Al frame was approximately 3% to 18% higher at all heights than that of paper tube. This higher dose rate is attributed to beta-rays emitted from the beta-minus decay of ^28^Al influencing the BeO ceramic TLD. The thermal neutron capture cross section of ^27^Al in the Al frame is 230.3 mb [22], and the ^28^Al produced in this reaction undergoes beta-minus decay to ^28^Si with a half-life of 2.24 min. In the present study, unlike the BeO powder TLD, which was encapsulated in a quartz glass tube, the BeO ceramic TLD was covered only with a thin soft polyvinyl chloride light shielding bag and was placed directly in the Al frame. Thus, the dose might include the effect of beta-rays produced by ^28^Al decay. Figure 12b shows a comparison of the gamma-ray dose rates when the BeO powder TLD is placed in the Al frame and in the paper tube. As previously mentioned, for the BeO ceramic TLD, the dose rate was approximately 3% to 18% higher at all heights when placed in the Al frame than when placed in the paper tube. However, the BeO powder TLD encapsulated in a quartz glass tube had a dose rate of approximately − 3% to 7% relative to the paper tube when placed in the Al frame. The smaller difference in dose rate between the two compared with the corresponding difference observed for the BeO ceramic TLD suggests that the effect of beta-rays produced by ^28^Al decay in the BeO powder TLD was reduced by the quartz glass.

### Comparison of thermal neutron flux distribution and gamma-ray dose rate distribution

**Fig. 13:**
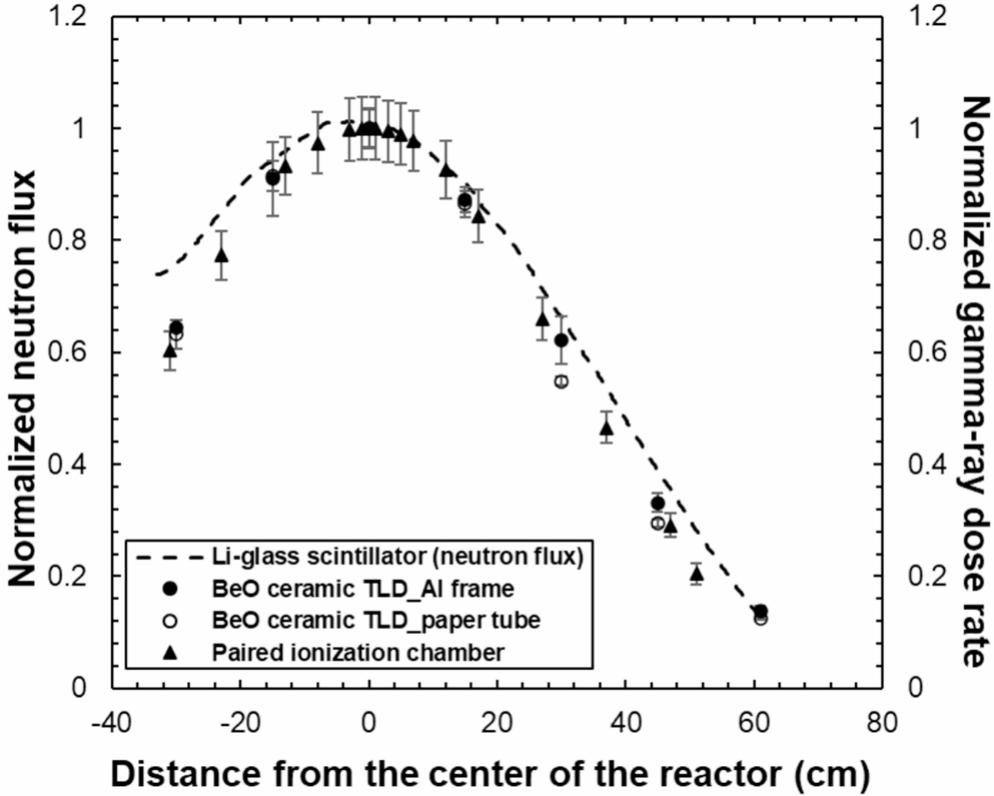
Comparison of the thermal neutron flux distribution measured using a Li-glass scintillator and the gamma-ray dose rate distributions measured using the BeO ceramic TLD and a paired ionization chamber

Figure 13 shows a comparison of thermal neutron flux distribution measured using a Li-glass scintillator and the gamma-ray dose rate distributions measured using a BeO ceramic TLD and paired ionization chamber inside the irradiation hole. The thermal neutron flux and gamma-ray dose rates were normalized to the value at the center of the reactor, which was set to 1. In addition, the thermal neutron flux distribution was smoothed.

The height dependence of the gamma-ray dose rate distribution showed a comparable result between the BeO ceramic TLD and the paired ionization chamber, and both exhibit a symmetric distribution. Compared with the thermal neutron flux distribution, both distributions demonstrated a similar tendency in height dependence above − 15 cm from the center of the reactor. However, the slope of the thermal neutron flux distribution near the bottom of the irradiation hole is gentle compared with the slope of the gamma-ray dose rate distribution. A difference of approximately 13% in thermal neutron flux was observed between − 33 cm and 33 cm from the center of the reactor. Kitamura et al. [13] have clarified that, in this irradiation hole, the neutron flux distribution, when the entire central stringer is housed, shows a contrasting distribution. Therefore, this difference in trend is suggested to be caused by the fact that neutrons are reflected by the central stringer left at the bottom of the irradiation hole, whereas gamma-rays are less affected by the interaction with the graphite. Thus, the results demonstrate that, when part of the central stringer is pulled out and irradiated, the irradiation field near the remaining central stringer is more affected by thermal neutrons.

## Conclusions

In this study, the height dependence of the gamma-ray dose rate distribution measured using a BeO ceramic TLD and a BeO powder TLD and the height dependence of the thermal neutron flux distribution measured using Li-glass scintillators in the irradiation hole of UTR-KINKI were evaluated. In the measurement of the gamma-ray dose rate distribution, the samples for irradiation were placed on an Al frame and on a paper tube. The results suggested that, when the irradiation sample is directly placed on the Al frame, the beta-rays generated by the decay of ^28^Al could lead to an overestimation of the dose. The present study is the first report of the gamma-ray dose rate distribution measured with a BeO ceramic TLD, where the gamma-ray dose was measured without considering the influence of neutrons. At the center of the reactor, the dose rate was approximately 24 cGy h^− 1^. The height dependence of the thermal neutron flux distribution was suggested to be influenced by the remaining central stringer in the irradiation hole, and the results clarified that the number of thermal neutrons increases near the remaining central stringer.

This study provides information on the irradiation field characteristics of the irradiation hole as a foundation for numerous studies conducted at UTR-KINKI. In addition, the gamma-ray dose measurement method using a BeO ceramic TLD, which is not sensitive to neutrons, is not affected by variations in the irradiation field. Therefore, the proposed method is expected to become the standard method for gamma-ray dose measurements in neutron fields in the future.
